# Correction: Fan et al. Comparing the Effects of AI-Assisted and Traditional Exercise on Physical Health Outcomes in Older Adults: A Systematic Review and Meta-Analysis. *Healthcare* 2025, *13*, 2999

**DOI:** 10.3390/healthcare14040428

**Published:** 2026-02-09

**Authors:** Sijing Fan, Xin Tan, Hongyun Zheng, Yicong Cui, Xiaotong Du, Boqiao Huang, Jingzhan Ren, Xinming Ye, Wen Fang

**Affiliations:** 1 School of Sports, Science and Engineering, East China University of Science and Technology, 130 Meilong Road, Xuhui District, Shanghai 200237, China; y30231582@mail.ecust.edu.cn (S.F.); y30241614@mail.ecust.edu.cn (H.Z.); tong20000529@gmail.com (X.D.); jkyjs@mail.ecust.edu.cn (X.Y.); 2 College of Smart Materials and Future Energy, Fudan University, 220 Handan Road, Yangpu District, Shanghai 200433, China; xtan21@m.fudan.edu.cn; 3 College of Engineering and Design, Hunan Normal University, 36 Lushan Road, Yuelu District, Changsha 410081, China; 4 Division of Sports Science and Physical Education, Tsinghua University, No. 30 Shuangqing Road, Haidian District, Beijing 100084, China; cyc23@mails.tsinghua.edu.cn; 5 China Japan Union Hospital, Jilin University, No. 2699 Qianjin Street, Changchun 130012, China; huangbq9922@mails.jlu.edu.cn; 6 School of Business, East China University of Science and Technology, 130 Meilong Road, Xuhui District, Shanghai 200237, China; y30231329@mail.ecust.edu.cn

**Missing Funding**

In the original publication [1], the funders, the Shanghai Education Science Planning Project (No. A2025003), the Shanghai Education Commission’s Scientific and Technological Innovation Project (No. L100-2-24109), and a sub-project of the collaborative research project between Anhui Sanlian Group and East China University of Science and Technology, Anhui Sanlian College (No. L110-72581), were not included. The corrected Funding statement appears below:

**Funding:** This research was supported by the Shanghai Education Science Planning Project (No. A2025003), the Shanghai Education Commission’s Scientific and Technological Innovation Project (No. L100-2-24109), and a sub-project of the collaborative research project between Anhui Sanlian Group and East China University of Science and Technology, Anhui Sanlian College (No. L110-72581).

The authors state that the scientific conclusions are unaffected. This correction was approved by the Academic Editor. The original publication has also been updated.
