# Supplementary material for: Comparing the Effects of AI-Assisted and Traditional Exercise on Physical Health Outcomes in Older Adults: A Systematic Review and Meta-Analysis
Source: Healthcare (Basel). 2025 Nov 21;13(23):2999. doi: 10.3390/healthcare13232999 (PMC12692026; doi:10.3390/healthcare13232999)
Supplement: Supplementary file 1 [file healthcare-13-02999-s001.zip › S1 Search Strategies for Five Databases.pdf]

Comparing the Effects of AI-Assisted and Traditional Exercise on Physical Health Outcomes in Older Adults: A Systematic Review and Meta-Analysis

Supplementary Notes

1. Search strategies for different databases

Pubmed:

((“Artificial Intelligence”[Mesh] OR “Artificial Intelligence”[tiab] OR “AI”[tiab] OR “machine learning”[tiab] OR “Virtual Reality”[Mesh] OR “virtual reality”[tiab] OR “smart technology”[tiab] OR “digital tools”[tiab]) AND (“Exercise”[Mesh] OR “exercise”[tiab] OR “physical activity”[tiab] OR “sports”[tiab] OR “fitness”[tiab] OR “training”[tiab]) AND (“Cognition”[Mesh] OR “cognitive function”[tiab] OR “cognitive improvement”[tiab] OR “Memory”[Mesh] OR “Attention”[Mesh] OR “executive function”[tiab] OR “Reaction Time”[Mesh] OR “brain health”[tiab]) AND (“Aged”[Mesh] OR “older adults”[tiab] OR “elderly”[tiab] OR “aging population”[tiab] OR “seniors”[tiab]))

Embase:

(('artificial intelligence'/exp OR 'artificial intelligence' OR 'machine intelligence':ti,ab,kw OR 'artificial intelligence':ti,ab,kw) AND ('aged'/exp OR 'aged' OR 'aged patient':ti,ab,kw OR 'aged people':ti,ab,kw OR 'aged person':ti,ab,kw OR 'aged subject':ti,ab,kw OR 'elderly':ti,ab,kw OR 'elderly patient':ti,ab,kw OR 'elderly people':ti,ab,kw OR 'elderly person':ti,ab,kw OR 'elderly subject':ti,ab,kw OR 'senior citizen':ti,ab,kw OR 'senium':ti,ab,kw OR 'aged or old':ti,ab,kw OR elder:ti,ab,kw OR senior:ti,ab,kw OR elderly:ti,ab,kw)AND ('randomized controlled trial':it OR randomized:ti,ab,kw OR placebo:ti,ab,kw)AND ('exercise'/exp OR 'exercise' OR 'biometric exercise':ti,ab,kw OR 'effort':ti,ab,kw OR 'exercise capacity':ti,ab,kw OR 'exercise performance':ti,ab,kw OR 'exercise training':ti,ab,kw OR 'exertion':ti,ab,kw OR 'fitness training':ti,ab,kw OR 'fitness workout':ti,ab,kw OR 'physical conditioning, human':ti,ab,kw OR 'physical effort':ti,ab,kw OR 'physical exercise':ti,ab,kw OR 'physical exertion':ti,ab,kw OR 'physical work-out':ti,ab,kw OR 'physical workout':ti,ab,kw OR 'exercise':ti,ab,kw))AND [embase]/lim

Cochrane Library

("artificial intelligence":ti,ab,kw OR "AI":ti,ab,kw OR "companion AI":ti,ab,kw OR "socially assistive AI":ti,ab,kw OR "companion robot":ti,ab,kw OR "social robot":ti,ab,kw OR "elderly assistive AI":ti,ab,kw OR "wearable technology":ti,ab,kw OR "smart wearable":ti,ab,kw OR "AI companion wearable":ti,ab,kw OR "AI companion":ti,ab,kw OR "assistive device":ti,ab,kw OR "digital":ti,ab,kw OR "robotic assistant":ti,ab,kw OR "intelligent personal assistant":ti,ab,kw OR "care robot":ti,ab,kw OR "cognitive robot":ti,ab,kw OR "cognitive assistant":ti,ab,kw OR "smart speaker":ti,ab,kw OR "AI-driven technology":ti,ab,kw OR "virtual assistant":ti,ab,kw OR "health technology":ti,ab,kw OR "digital health tool":ti,ab,kw OR "assistive robotics":ti,ab,kw OR "smart home device":ti,ab,kw OR "voice assistant":ti,ab,kw OR "intelligent agent":ti,ab,kw OR "AI system":ti,ab,kw OR "virtual care":ti,ab,kw OR "telehealth":ti,ab,kw OR "telemedicine":ti,ab,kw OR "ambient assisted living":ti,ab,kw OR "healthcare robot":ti,ab,kw OR "personal healthcare":ti,ab,kw OR "personal healthcare device":ti,ab,kw OR "AI health assistant":ti,ab,kw OR "smart caregiving":ti,ab,kw OR "elder care technology":ti,ab,kw OR "intelligent health monitoring":ti,ab,kw)

Web of science

TS = (("artificial intelligence" OR "machine intelligence")
AND ("aged" OR "elderly" OR "senior" OR "older adults" OR "aged people" OR "aged person" OR "elderly people" OR "elderly person" OR "aged patient" OR "elderly patient" OR "aged subject" OR "elderly subject" OR "senior citizen" OR "senium") AND ("exercise" OR "physical exercise" OR "exercise training" OR "fitness training" OR "fitness workout" OR "physical conditioning" OR "physical exertion" OR "physical effort" OR "exercise

performance" OR "exercise capacity" OR "exercise program" OR "rehabilitation" OR "motor training" OR "physical therapy" OR "movement training") AND ("randomized controlled trial" OR "randomized" OR "RCT" OR "controlled clinical trial" OR "placebo"))

Scoups:

(TITLE-ABS-KEY ("artificial intelligence" OR "machine intelligence") AND TITLE-ABS-KEY (aged OR elderly OR "older adults" OR "aged people" OR "aged person" OR "elderly people" OR "elderly person" OR "aged patient" OR "elderly patient" OR "aged subject" OR "elderly subject" OR "senior citizen" OR senior) AND TITLE-ABS-KEY (exercise OR "physical exercise" OR "exercise training" OR "fitness training" OR "fitness workout" OR "physical conditioning" OR "physical exertion" OR "physical effort" OR "exercise performance" OR "exercise capacity" OR rehabilitation OR "motor training" OR "physical therapy" OR "movement training") AND TITLE-ABS-KEY ("randomized controlled trial" OR randomized OR RCT OR "controlled clinical trial" OR placebo))
