# Supplementary material for: Comparing the Effects of AI-Assisted and Traditional Exercise on Physical Health Outcomes in Older Adults: A Systematic Review and Meta-Analysis
Source: Healthcare (Basel). 2025 Nov 21;13(23):2999. doi: 10.3390/healthcare13232999 (PMC12692026; doi:10.3390/healthcare13232999)
Supplement: Supplementary file 1 [file healthcare-13-02999-s001.zip › S4.Data _ AI VS Traditional NMA/a/I2 τ2.pdf]

```
  ___  ____  ____  ____  ____ ®
 /__    /   ____/   /   ____/      18.0
___/   /   /___/   /   /___/       MP—Parallel Edition

 Statistics and Data Science       Copyright 1985-2023 StataCorp LLC
                                   StataCorp
                                   4905 Lakeway Drive
                                   College Station, Texas 77845 USA
                                   800-STATA-PC        https://www.stata.com
                                   979-696-4600        stata@stata.com

Stata license: Single-user 2-core  perpetual
Serial number: 501806366047
  Licensed to:


Notes:
      1. Unicode is supported; see help unicode_advice.
      2. More than 2 billion observations are allowed; see help obs_advice.
      3. Maximum number of variables is set to 5,000 but can be increased; see help set_maxvar.

1 . *(7 variables, 36 observations pasted into data editor)

2 . meta set smd se
(18 missing values generated)

Meta-analysis setting information

 Study information
    No. of studies: 18
       Study label: Generic
        Study size: N/A

       Effect size
              Type: <generic>
             Label: Effect size
          Variable: smd

         Precision
         Std. err.: se_smd
                CI: [_meta_cil, _meta_ciu]
          CI level: 95%

  Model and method
             Model: Random effects
            Method: REML

3 . meta summarize, random(dl)

  Effect-size label: Effect size
        Effect size: smd
          Std. err.: se_smd
```

```
Meta-analysis summary                     Number of studies =     18
Random-effects model                      Heterogeneity:
Method: DerSimonian–Laird                             tau2 =  0.2449
                                                    I2 (%) =   63.45
                                                        H2 =    2.74
```

Study	Effect size	[95% conf.	interval]	% weight
Study 2	**0.130**	**-0.885**	**1.145**	**4.27**
Study 4	**0.670**	**-0.092**	**1.432**	**5.52**
Study 6	**0.510**	**-0.245**	**1.265**	**5.57**
Study 8	**1.160**	**0.360**	**1.960**	**5.32**
Study 10	**0.530**	**-0.154**	**1.214**	**5.97**
Study 12	**0.590**	**-0.098**	**1.278**	**5.95**
Study 14	**0.240**	**-0.436**	**0.916**	**6.01**
Study 16	**2.340**	**1.542**	**3.138**	**5.33**
Study 18	**0.880**	**0.233**	**1.527**	**6.19**
Study 20	**1.410**	**0.483**	**2.337**	**4.67**
Study 22	**0.000**	**-0.806**	**0.806**	**5.29**
Study 24	**0.041**	**-0.553**	**0.635**	**6.50**
Study 26	**1.000**	**0.308**	**1.692**	**5.92**
Study 28	**0.930**	**0.244**	**1.616**	**5.96**
Study 30	**0.130**	**-0.462**	**0.722**	**6.51**
Study 32	**2.420**	**1.287**	**3.553**	**3.78**
Study 34	**0.250**	**-0.493**	**0.993**	**5.63**
Study 36	**0.430**	**-0.319**	**1.179**	**5.60**
theta	**0.714**	**0.424**	**1.004**	

```
Test of theta = 0: z = 4.83                        Prob > |z| = 0.0000
Test of homogeneity: Q = chi2(17) = 46.52            Prob > Q = 0.0001

4 . meta summarize, subgroup(t)

  Effect-size label: Effect size
        Effect size: smd
          Std. err.: se_smd

Subgroup meta-analysis summary            Number of studies =     18
Random-effects model
Method: REML
Group: t
```

Study	Effect size	[95% conf.	interval]	% weight
Group: 1				
Study 2	**0.130**	**-0.885**	**1.145**	**4.30**
Study 12	**0.590**	**-0.098**	**1.278**	**5.93**
Study 16	**2.340**	**1.542**	**3.138**	**5.34**
Study 18	**0.880**	**0.233**	**1.527**	**6.16**
Study 20	**1.410**	**0.483**	**2.337**	**4.70**
Study 26	**1.000**	**0.308**	**1.692**	**5.91**

```
        Study 28 |         0.930       0.244       1.616      5.95
        Study 30 |         0.130      -0.462       0.722      6.48
        Study 32 |         2.420       1.287       3.553      3.83
                 |
           theta |         1.050       0.536       1.563
-----------------+-------------------------------------------------
Group: 3         |
        Study  4 |         0.670      -0.092       1.432      5.53
        Study  6 |         0.510      -0.245       1.265      5.57
        Study  8 |         1.160       0.360       1.960      5.33
        Study 10 |         0.530      -0.154       1.214      5.96
        Study 14 |         0.240      -0.436       0.916      6.00
        Study 22 |         0.000      -0.806       0.806      5.30
        Study 24 |         0.041      -0.553       0.635      6.46
        Study 34 |         0.250      -0.493       0.993      5.63
        Study 36 |         0.430      -0.319       1.179      5.60
                 |
           theta |         0.395       0.155       0.636
-----------------+-------------------------------------------------
Overall          |
           theta |         0.716       0.420       1.011
-------------------------------------------------------------------

Heteregeneity summary
---------------------------------------------------------------------------
          Group |    df        Q   P > Q        tau2      % I2        H2
----------------+----------------------------------------------------------
              1 |     8    30.07   0.000       0.453     75.30      4.05
              3 |     8     6.90   0.548       0.000      0.00      1.00
----------------+----------------------------------------------------------
        Overall |    17    46.52   0.000       0.260     64.82      2.84
---------------------------------------------------------------------------
Test of group differences: Q_b = chi2(1) = 5.12           Prob > Q_b = 0.024

5 . meta regress i.t, random(dl)
note: 4.t identifies no observations in the sample.
note: 5.t identifies no observations in the sample.

  Effect-size label: Effect size
        Effect size: smd
          Std. err.: se_smd

Random-effects meta-regression                      Number of obs  =        18
Method: DerSimonian-Laird                           Residual heterogeneity:
                                                                tau2 =   .1859
                                                              I2 (%) =   56.72
                                                                  H2 =    2.31
                                                       R-squared (%) =   24.07
                                                    Wald chi2(1)   =      4.94
                                                    Prob > chi2    =    0.0262
------------------------------------------------------------------------------
     _meta_es | Coefficient  Std. err.      z    P>|z|     [95% conf. interval]
--------------+---------------------------------------------------------------
            t |
```

```
           3 |  -.6058471   .2725562    -2.22   0.026    -1.140048   -.0716467
           4 |          0  (empty)
           5 |          0  (empty)
             |
       _cons |   1.020229   .1957126     5.21   0.000     .6366395    1.403819
-----------------------------------------------------------------------------
Test of residual homogeneity: Q_res = chi2(16) = 36.97   Prob > Q_res = 0.0021

6 .
```
