# Supplementary material for: Comparing the Effects of AI-Assisted and Traditional Exercise on Physical Health Outcomes in Older Adults: A Systematic Review and Meta-Analysis
Source: Healthcare (Basel). 2025 Nov 21;13(23):2999. doi: 10.3390/healthcare13232999 (PMC12692026; doi:10.3390/healthcare13232999)
Supplement: Supplementary file 1 [file healthcare-13-02999-s001.zip › S4.Data _ AI VS Traditional NMA/b/I2 τ2.pdf]

```
  ___  ____  ____  ____  ____ ®
 /__    /   ____/   /   ____/      18.0
___/   /   /___/   /   /___/       MP—Parallel Edition

 Statistics and Data Science       Copyright 1985-2023 StataCorp LLC
                                   StataCorp
                                   4905 Lakeway Drive
                                   College Station, Texas 77845 USA
                                   800-STATA-PC        https://www.stata.com
                                   979-696-4600        stata@stata.com

Stata license: Single-user 2-core  perpetual
Serial number: 501806366047
  Licensed to:


Notes:
      1. Unicode is supported; see help unicode_advice.
      2. More than 2 billion observations are allowed; see help obs_advice.
      3. Maximum number of variables is set to 5,000 but can be increased; see help set_maxvar.

1 . *(7 variables, 28 observations pasted into data editor)

2 . meta set smd se
(14 missing values generated)

Meta-analysis setting information

 Study information
    No. of studies: 14
       Study label: Generic
        Study size: N/A

       Effect size
              Type: <generic>
             Label: Effect size
          Variable: smd

         Precision
          Std. err.: se_smd
                CI: [_meta_cil, _meta_ciu]
          CI level: 95%

  Model and method
             Model: Random effects
            Method: REML

3 . meta summarize, random(dl)

  Effect-size label: Effect size
        Effect size: smd
          Std. err.: se_smd

Meta-analysis summary                     Number of studies =     14
Random-effects model                      Heterogeneity:
Method: DerSimonian-Laird                             tau2 =  0.0000
                                                    I2 (%) =    0.00
                                                        H2 =    1.00
```

Study	Effect size	[95% conf.	interval]	% weight
Study 2	0.270	-0.426	0.966	7.58
Study 4	0.410	-0.615	1.435	3.49
Study 6	0.240	-0.603	1.083	5.17
Study 8	0.730	-0.036	1.496	6.25
Study 10	0.230	-0.446	0.906	8.03
Study 12	0.080	-0.594	0.754	8.08
Study 14	0.850	-0.022	1.722	4.83
Study 16	0.790	-0.078	1.658	4.87
Study 18	0.470	-0.524	1.464	3.72
Study 20	0.060	-0.546	0.666	10.01
Study 22	0.085	-0.487	0.657	11.21
Study 24	0.730	0.120	1.340	9.88
Study 26	0.290	-0.302	0.882	10.48
Study 28	0.390	-0.367	1.147	6.41
theta	0.358	0.166	0.549	

```
Test of theta = 0: z = 3.66                      Prob > |z| = 0.0003
```

```
  Test of homogeneity: Q = chi2(13) = 7.36           Prob > Q = 0.8828

4 .
```
