# Supplementary material for: Comparing the Effects of AI-Assisted and Traditional Exercise on Physical Health Outcomes in Older Adults: A Systematic Review and Meta-Analysis
Source: Healthcare (Basel). 2025 Nov 21;13(23):2999. doi: 10.3390/healthcare13232999 (PMC12692026; doi:10.3390/healthcare13232999)
Supplement: Supplementary file 1 [file healthcare-13-02999-s001.zip › S4.Data _ AI VS Traditional NMA/c/I2 τ2 new.pdf]

```
  ___  ____  ____  ____  ____ ®
 /__    /   ____/   /   ____/      18.0
___/   /   /___/   /   /___/       MP—Parallel Edition

 Statistics and Data Science       Copyright 1985-2023 StataCorp LLC
                                   StataCorp
                                   4905 Lakeway Drive
                                   College Station, Texas 77845 USA
                                   800-STATA-PC        https://www.stata.com
                                   979-696-4600        stata@stata.com

Stata license: Single-user 2-core  perpetual
Serial number: 501806366047
  Licensed to:


Notes:
      1. Unicode is supported; see help unicode_advice.
      2. More than 2 billion observations are allowed; see help obs_advice.
      3. Maximum number of variables is set to 5,000 but can be increased; see help set_maxvar.

1 . *(7 variables, 18 observations pasted into data editor)

2 . meta set smd se
(9 missing values generated)

Meta-analysis setting information

 Study information
    No. of studies: 9
       Study label: Generic
        Study size: N/A

       Effect size
              Type: <generic>
             Label: Effect size
          Variable: smd

         Precision
         Std. err.: se_smd
                CI: [_meta_cil, _meta_ciu]
          CI level: 95%

  Model and method
             Model: Random effects
            Method: REML

3 . meta summarize, random(dl)

  Effect-size label: Effect size
        Effect size: smd
          Std. err.: se_smd

Meta-analysis summary                     Number of studies =      9
Random-effects model                      Heterogeneity:
Method: DerSimonian-Laird                              tau2 =  0.0000
                                                     I2 (%) =    0.00
                                                         H2 =    1.00
```

Study	Effect size	[95% conf.	interval]	% weight
Study 2	0.380	-0.439	1.199	7.29
Study 4	0.130	-0.681	0.941	7.44
Study 6	0.212	-0.531	0.955	8.87
Study 8	0.510	0.071	0.949	25.40
Study 10	0.308	-0.529	1.145	6.99
Study 12	0.720	-0.142	1.582	6.58
Study 14	0.610	-0.233	1.453	6.89
Study 16	1.540	0.603	2.477	5.58
Study 18	0.396	-0.047	0.839	24.95
theta	0.481	0.260	0.703	

```
Test of theta = 0: z = 4.26                    Prob > |z| = 0.0000
Test of homogeneity: Q = chi2(8) = 6.90          Prob > Q = 0.5478

4 . meta summarize, subgroup(t)

  Effect-size label: Effect size
        Effect size: smd
          Std. err.: se_smd

Subgroup meta-analysis summary            Number of studies =      9
Random-effects model
Method: REML
Group: t
```

Study	Effect size	[95% conf.	interval]	% weight
Group: 1				

Study 2	**0.380**	**−0.439**	**1.199**	**7.29**
Study 4	**0.130**	**−0.681**	**0.941**	**7.44**
Study 8	**0.510**	**0.071**	**0.949**	**25.40**
Study 14	**0.610**	**−0.233**	**1.453**	**6.89**
Study 16	**1.540**	**0.603**	**2.477**	**5.58**
theta	**0.561**	**0.256**	**0.866**	
Group: 2				
Study 10	**0.308**	**−0.529**	**1.145**	**6.99**
Study 12	**0.720**	**−0.142**	**1.582**	**6.58**
theta	**0.508**	**−0.093**	**1.108**	
Group: 3				
Study 6	**0.212**	**−0.531**	**0.955**	**8.87**
Study 18	**0.396**	**−0.047**	**0.839**	**24.95**
theta	**0.348**	**−0.033**	**0.728**	
Overall				
theta	**0.481**	**0.260**	**0.703**	

Heterogeneity summary

Group	df	Q	P > Q	tau2	% I2	H2
1	**4**	**5.53**	**0.237**	**0.000**	**0.00**	**1.00**
2	**1**	**0.45**	**0.502**	**0.000**	**0.00**	**1.00**
3	**1**	**0.17**	**0.677**	**0.000**	**0.00**	**1.00**
Overall	**8**	**6.90**	**0.548**	**0.000**	**0.00**	**1.00**

Test of group differences: Q_b = chi2(**2**) = **0.74** Prob > Q_b = **0.691**

5 .
