# Supplementary material for: Comparing the Effects of AI-Assisted and Traditional Exercise on Physical Health Outcomes in Older Adults: A Systematic Review and Meta-Analysis
Source: Healthcare (Basel). 2025 Nov 21;13(23):2999. doi: 10.3390/healthcare13232999 (PMC12692026; doi:10.3390/healthcare13232999)
Supplement: Supplementary file 1 [file healthcare-13-02999-s001.zip › S4.Data _ AI VS Traditional NMA/c/I2 τ2.pdf]

```
  ___  ____  ____  ____  ____ ®
 /__    /   ____/   /   ____/      18.0
___/   /   /___/   /   /___/       MP—Parallel Edition

 Statistics and Data Science       Copyright 1985-2023 StataCorp LLC
                                   StataCorp
                                   4905 Lakeway Drive
                                   College Station, Texas 77845 USA
                                   800-STATA-PC        https://www.stata.com
                                   979-696-4600        stata@stata.com

Stata license: Single-user 2-core  perpetual
Serial number: 501806366047
  Licensed to:


Notes:
      1. Unicode is supported; see help unicode_advice.
      2. More than 2 billion observations are allowed; see help obs_advice.
      3. Maximum number of variables is set to 5,000 but can be increased; see help set_maxvar.

1 . *(7 variables, 18 observations pasted into data editor)

2 . meta set smd se
(9 missing values generated)

Meta-analysis setting information

 Study information
    No. of studies: 9
       Study label: Generic
        Study size: N/A

       Effect size
              Type: &lt;generic&gt;
             Label: Effect size
          Variable: smd

         Precision
          Std. err.: se_smd
                CI: [_meta_cil, _meta_ciu]
          CI level: 95%

  Model and method
             Model: Random effects
            Method: REML

3 . meta summarize, random(dl)

  Effect-size label: Effect size
        Effect size: smd
          Std. err.: se_smd

Meta-analysis summary                    Number of studies =      9
Random-effects model                     Heterogeneity:
Method: DerSimonian-Laird                            tau2 =  0.1803
                                                   I2 (%) =   59.81
                                                       H2 =    2.49
```

Study	Effect size	[95% conf.	interval]	% weight
Study 2	**-0.380**	**-1.199**	**0.439**	**10.03**
Study 4	**-0.130**	**-0.941**	**0.681**	**10.13**
Study 6	**-0.030**	**-0.773**	**0.713**	**11.00**
Study 8	**0.510**	**0.071**	**0.949**	**15.46**
Study 10	**-0.100**	**-0.937**	**0.737**	**9.82**
Study 12	**-0.720**	**-1.582**	**0.142**	**9.53**
Study 14	**-0.646**	**-1.491**	**0.199**	**9.73**
Study 16	**-1.453**	**-2.378**	**-0.528**	**8.84**
Study 18	**0.000**	**-0.439**	**0.439**	**15.46**
theta	**-0.245**	**-0.615**	**0.124**	

```
Test of theta = 0: z = -1.30                  Prob > |z| = 0.1934
Test of homogeneity: Q = chi2(8) = 19.91        Prob > Q = 0.0107

4 . meta summarize, subgroup(t)

  Effect-size label: Effect size
        Effect size: smd
          Std. err.: se_smd

Subgroup meta-analysis summary           Number of studies =      9
Random-effects model
Method: REML
Group: t
```

Study	Effect size	[95% conf.	interval]	% weight
Group: 1				

Study 2	−0.380	−1.199	0.439	10.02
Study 4	−0.130	−0.941	0.681	10.12
Study 8	0.510	0.071	0.949	15.51
Study 14	−0.646	−1.491	0.199	9.71
Study 16	−1.453	−2.378	−0.528	8.81
theta	−0.354	−1.006	0.299	
Group: 2				
Study 10	−0.100	−0.937	0.737	9.81
Study 12	−0.720	−1.582	0.142	9.51
theta	−0.401	−1.008	0.206	
Group: 3				
Study 6	−0.030	−0.773	0.713	10.99
Study 18	0.000	−0.439	0.439	15.51
theta	−0.008	−0.386	0.370	
Overall				
theta	−0.244	−0.612	0.123	

Heterogeneity summary

Group	df	Q	P > Q	tau2	% I2	H2
1	4	17.65	0.001	0.400	74.35	3.90
2	1	1.02	0.312	0.004	2.20	1.02
3	1	0.00	0.946	0.000	0.00	1.00
Overall	8	19.91	0.011	0.177	59.35	2.46

Test of group differences: Q_b = chi2(**2**) = **1.57** Prob > Q_b = **0.457**

5 .
