# Supplementary material for: Comparing the Effects of AI-Assisted and Traditional Exercise on Physical Health Outcomes in Older Adults: A Systematic Review and Meta-Analysis
Source: Healthcare (Basel). 2025 Nov 21;13(23):2999. doi: 10.3390/healthcare13232999 (PMC12692026; doi:10.3390/healthcare13232999)
Supplement: Supplementary file 1 [file healthcare-13-02999-s001.zip › S4.Data _ AI VS Traditional NMA/d/I2 τ2 .pdf]

```
  ___  ____  ____  ____  ____ ®
 /__    /   ____/   /   ____/      18.0
___/   /   /___/   /   /___/       MP—Parallel Edition

 Statistics and Data Science       Copyright 1985-2023 StataCorp LLC
                                   StataCorp
                                   4905 Lakeway Drive
                                   College Station, Texas 77845 USA
                                   800-STATA-PC        https://www.stata.com
                                   979-696-4600        stata@stata.com

Stata license: Single-user 2-core  perpetual
Serial number: 501806366047
  Licensed to:


Notes:
      1. Unicode is supported; see help unicode_advice.
      2. More than 2 billion observations are allowed; see help obs_advice.
      3. Maximum number of variables is set to 5,000 but can be increased; see help set_maxvar.

1 . *(7 variables, 18 observations pasted into data editor)

2 . meta set smd se
  (9 missing values generated)

  Meta-analysis setting information

    Study information
       No. of studies: 9
          Study label: Generic
           Study size: N/A

          Effect size
                 Type: <generic>
                Label: Effect size
             Variable: smd

            Precision
            Std. err.: se_smd
                   CI: [_meta_cil, _meta_ciu]
             CI level: 95%

     Model and method
                Model: Random effects
               Method: REML

3 . meta summarize, random(dl)

    Effect-size label: Effect size
          Effect size: smd
            Std. err.: se_smd

  Meta-analysis summary                  Number of studies =      9
  Random-effects model                   Heterogeneity:
  Method: DerSimonian-Laird                          tau2 =  0.1185
                                                   I2 (%) =   46.57
                                                       H2 =    1.87
```

Study	Effect size	[95% conf.	interval]	% weight
Study 2	**0.630**	**-0.129**	**1.389**	**10.93**
Study 4	**0.110**	**-0.631**	**0.851**	**11.22**
Study 6	**0.220**	**-0.476**	**0.916**	**12.00**

```
       Study 8 |      0.440     -0.262      1.142     11.89
      Study 10 |     -0.410     -1.435      0.615      7.48
      Study 12 |     -0.550     -1.377      0.277      9.89
      Study 14 |      0.040     -0.771      0.851     10.12
      Study 16 |     -1.069     -1.949     -0.189      9.16
      Study 18 |      0.420     -0.023      0.863     17.30
---------------+--------------------------------------------
         theta |      0.054     -0.282      0.389
---------------+--------------------------------------------
Test of theta = 0: z = 0.31                 Prob > |z| = 0.7542
Test of homogeneity: Q = chi2(8) = 14.97       Prob > Q = 0.0597
```

```
4 . meta summarize, subgroup(t)

  Effect-size label: Effect size
        Effect size: smd
          Std. err.: se_smd

Subgroup meta-analysis summary          Number of studies =      9
Random-effects model
Method: REML
Group: t
```

Study	Effect size	[95% conf.	interval]	% weight
**Group: 1**				
Study 10	-0.410	-1.435	0.615	7.48
Study 12	-0.550	-1.377	0.277	9.89
Study 14	0.040	-0.771	0.851	10.12
Study 16	-1.069	-1.949	-0.189	9.16
theta	-0.484	-0.960	-0.008	
**Group: 2**				
Study 2	0.630	-0.129	1.389	10.93
Study 4	0.110	-0.631	0.851	11.22
Study 6	0.220	-0.476	0.916	12.00
Study 8	0.440	-0.262	1.142	11.89
theta	0.345	-0.016	0.707	
**Group: 3**				
Study 18	0.420	-0.023	0.863	17.30
theta	0.420	-0.023	0.863	
**Overall**				
theta	0.054	-0.282	0.389	

Heterogeneity summary

Group	df	Q	P > Q	tau2	% I2	H2
1	3	3.34	0.342	0.036	15.12	1.18
2	3	1.12	0.771	0.000	0.00	1.00
3	0	-0.00	.	0.000	.	.
Overall	8	14.97	0.060	0.118	46.56	1.87

```
Test of group differences: Q_b = chi2(2) = 9.36          Prob > Q_b = 0.009

5 . meta regress i.t, random(dl)
note: 4.t identifies no observations in the sample.
note: 5.t identifies no observations in the sample.
```

Effect-size label: Effect size
Effect size: **smd**
Std. err.: **se_smd**

Random-effects meta-regression
Method: DerSimonian–Laird

Number of obs = **9**
Residual heterogeneity:
tau2 = **0**
I2 (%) = **0.00**
H2 = **1.00**
R-squared (%) = **100.00**
Wald chi2(**2**) = **10.51**
Prob > chi2 = **0.0052**

_meta_es	Coefficient	Std. err.	z	P>|z|	[95% conf.	interval]
t						
2	**.8264977**	**.2895412**	**2.85**	**0.004**	**.2590075**	**1.393988**
3	**.901239**	**.3176658**	**2.84**	**0.005**	**.2786254**	**1.523853**
4	**0**	(empty)				
5	**0**	(empty)				
_cons	**–.481239**	**.2232389**	**–2.16**	**0.031**	**–.9187791**	**–.0436989**

Test of residual homogeneity: Q_res = chi2(**6**) = **4.47** Prob > Q_res = **0.6137**

6 .
