# Supplementary material for: Comparing the Effects of AI-Assisted and Traditional Exercise on Physical Health Outcomes in Older Adults: A Systematic Review and Meta-Analysis
Source: Healthcare (Basel). 2025 Nov 21;13(23):2999. doi: 10.3390/healthcare13232999 (PMC12692026; doi:10.3390/healthcare13232999)
Supplement: Supplementary file 1 [file healthcare-13-02999-s001.zip › S4.Data _ AI VS Traditional NMA/d/I2 τ2 new.pdf]

```
  ___  ____  ____  ____  ____ ®
 /__    /   ____/   /   ____/      18.0
___/   /   /___/   /   /___/       MP—Parallel Edition

 Statistics and Data Science       Copyright 1985-2023 StataCorp LLC
                                   StataCorp
                                   4905 Lakeway Drive
                                   College Station, Texas 77845 USA
                                   800-STATA-PC        https://www.stata.com
                                   979-696-4600        stata@stata.com

Stata license: Single-user 2-core  perpetual
Serial number: 501806366047
  Licensed to:


Notes:
      1. Unicode is supported; see help unicode_advice.
      2. More than 2 billion observations are allowed; see help obs_advice.
      3. Maximum number of variables is set to 5,000 but can be increased; see help set_maxvar.

1 . *(7 variables, 18 observations pasted into data editor)

2 . meta set smd se
  (9 missing values generated)

  Meta-analysis setting information

   Study information
      No. of studies: 9
         Study label: Generic
          Study size: N/A

         Effect size
                Type: <generic>
               Label: Effect size
            Variable: smd

           Precision
           Std. err.: se_smd
                  CI: [_meta_cil, _meta_ciu]
            CI level: 95%

    Model and method
               Model: Random effects
              Method: REML

3 . meta summarize, random(dl)

    Effect-size label: Effect size
          Effect size: smd
            Std. err.: se_smd

  Meta-analysis summary                     Number of studies =      9
  Random-effects model                      Heterogeneity:
  Method: DerSimonian–Laird                             tau2 =  0.0131
                                                      I2 (%) =    8.81
                                                          H2 =    1.10
```

Study	Effect size	[95% conf.	interval]	% weight
Study 2	0.630	-0.129	1.389	10.03
Study 4	0.110	-0.631	0.851	10.48
Study 6	0.220	-0.476	0.916	11.75

```
        Study 8 |      0.440     -0.262      1.142     11.57
       Study 10 |      0.405     -0.620      1.430      5.70
       Study 12 |     -0.550     -1.377      0.277      8.55
       Study 14 |      0.040     -0.771      0.851      8.86
       Study 16 |      1.058      0.178      1.938      7.61
       Study 18 |      0.420     -0.023      0.863     25.46
----------------+-------------------------------------------
          theta |      0.319      0.068      0.569
------------------------------------------------------------
Test of theta = 0: z = 2.49                  Prob > |z| = 0.0127
Test of homogeneity: Q = chi2(8) = 8.77        Prob > Q = 0.3618

4 . meta summarize, subgroup(t)

  Effect-size label: Effect size
        Effect size: smd
          Std. err.: se_smd

Subgroup meta-analysis summary          Number of studies =      9
Random-effects model
Method: REML
Group: t

------------------------------------------------------------
          Study |  Effect size    [95% conf. interval]  % weight
----------------+-------------------------------------------
Group: 1        |
       Study 10 |      0.405     -0.620      1.430      5.29
       Study 12 |     -0.550     -1.377      0.277      8.13
       Study 14 |      0.040     -0.771      0.851      8.45
       Study 16 |      1.058      0.178      1.938      7.18
                |
          theta |      0.219     -0.458      0.896
----------------+-------------------------------------------
Group: 2        |
        Study 2 |      0.630     -0.129      1.389      9.67
        Study 4 |      0.110     -0.631      0.851     10.13
        Study 6 |      0.220     -0.476      0.916     11.49
        Study 8 |      0.440     -0.262      1.142     11.30
                |
          theta |      0.345     -0.016      0.707
----------------+-------------------------------------------
Group: 3        |
       Study 18 |      0.420     -0.023      0.863     28.35
                |
          theta |      0.420     -0.023      0.863
----------------+-------------------------------------------
Overall         |
          theta |      0.322      0.086      0.558
------------------------------------------------------------

Heterogeneity summary
-----------------------------------------------------------------------
      Group |     df        Q   P > Q        tau2      % I2        H2
------------+----------------------------------------------------------
          1 |      3     7.11   0.068       0.274     57.69      2.36
          2 |      3     1.12   0.771       0.000      0.00      1.00
          3 |      0    -0.00       .       0.000         .         .
------------+----------------------------------------------------------
    Overall |      8     8.77   0.362       0.000      0.00      1.00
-----------------------------------------------------------------------
Test of group differences: Q_b = chi2(2) = 0.24        Prob > Q_b = 0.887

5 . meta regress i.t, random(dl)
note: 4.t identifies no observations in the sample.
note: 5.t identifies no observations in the sample.
```

```
  Effect-size label: Effect size
        Effect size: smd
          Std. err.: se_smd

Random-effects meta-regression                    Number of obs  =         9
Method: DerSimonian–Laird                         Residual heterogeneity:
                                                              tau2 =  .06054
                                                            I2 (%) =   27.14
                                                                H2 =    1.37
                                                     R-squared (%) =    0.00
                                                  Wald chi2(2)   =      0.31
                                                  Prob > chi2    =    0.8568

------------------------------------------------------------------------------
    _meta_es | Coefficient  Std. err.      z    P>|z|     [95% conf. interval]
-------------+----------------------------------------------------------------
           t |
          2  |   .1431251   .3383812     0.42   0.672    -.5200898    .8063401
          3  |   .2164846   .4206274     0.51   0.607      -.60793    1.040899
          4  |          0  (empty)
          5  |          0  (empty)
             |
       _cons |   .2035154    .255567     0.80   0.426    -.2973867    .7044174
------------------------------------------------------------------------------
Test of residual homogeneity: Q_res = chi2(6) =  8.24    Prob > Q_res = 0.2214

6 .
```
