# Supplementary material for: Comparing the Effects of AI-Assisted and Traditional Exercise on Physical Health Outcomes in Older Adults: A Systematic Review and Meta-Analysis
Source: Healthcare (Basel). 2025 Nov 21;13(23):2999. doi: 10.3390/healthcare13232999 (PMC12692026; doi:10.3390/healthcare13232999)
Supplement: Supplementary file 1 [file healthcare-13-02999-s001.zip › S4.Data _ AI VS Traditional NMA/e/I2 τ2 .pdf]

```
  ___  ____  ____  ____  ____ ®
 /__    /   ____/   /   ____/      18.0
___/   /   /___/   /   /___/       MP—Parallel Edition

 Statistics and Data Science       Copyright 1985-2023 StataCorp LLC
                                   StataCorp
                                   4905 Lakeway Drive
                                   College Station, Texas 77845 USA
                                   800-STATA-PC        https://www.stata.com
                                   979-696-4600        stata@stata.com

Stata license: Single-user 2-core  perpetual
Serial number: 501806366047
  Licensed to:


Notes:
      1. Unicode is supported; see help unicode_advice.
      2. More than 2 billion observations are allowed; see help obs_advice.
      3. Maximum number of variables is set to 5,000 but can be increased; see help set_maxvar.

1 . *(7 variables, 20 observations pasted into data editor)

2 . meta set smd se
  (10 missing values generated)

  Meta-analysis setting information

   Study information
      No. of studies: 10
         Study label: Generic
          Study size: N/A

         Effect size
                Type: <generic>
               Label: Effect size
            Variable: smd

           Precision
           Std. err.: se_smd
                  CI: [_meta_cil, _meta_ciu]
            CI level: 95%

    Model and method
               Model: Random effects
              Method: REML

3 . meta summarize, random(dl)

    Effect-size label: Effect size
          Effect size: smd
            Std. err.: se_smd

  Meta-analysis summary                     Number of studies =     10
  Random-effects model                      Heterogeneity:
  Method: DerSimonian-Laird                              tau2 =  0.0277
                                                       I2 (%) =   15.01
                                                           H2 =    1.18
```

Study	Effect size	[95% conf.	interval]	% weight
Study 2	0.134	-0.560	0.828	12.10
Study 4	0.947	0.108	1.786	8.78
Study 6	0.382	-0.437	1.201	9.14

Study	Effect size	[95% conf.	interval]	% weight
Study 8	**0.070**	**-0.910**	**1.050**	**6.66**
Study 10	**0.290**	**-0.692**	**1.272**	**6.64**
Study 12	**0.180**	**-0.426**	**0.786**	**15.03**
Study 14	**0.190**	**-0.416**	**0.796**	**15.03**
Study 16	**1.260**	**0.541**	**1.979**	**11.40**
Study 18	**0.510**	**-0.380**	**1.400**	**7.92**
Study 20	**1.010**	**0.079**	**1.941**	**7.31**
theta	**0.472**	**0.205**	**0.738**	

Test of theta = 0: z = **3.47** Prob > |z| = **0.0005**
Test of homogeneity: Q = chi2(**9**) = **10.59** Prob > Q = **0.3049**

```
4 . meta summarize, subgroup(t)
```

Effect-size label: Effect size
Effect size: **smd**
Std. err.: **se_smd**

Subgroup meta-analysis summary Number of studies = **10**
Random-effects model
Method: REML
Group: **t**

Study	Effect size	[95% conf.	interval]	% weight
Group: 1				
Study 6	**0.382**	**-0.437**	**1.201**	**9.24**
Study 10	**0.290**	**-0.692**	**1.272**	**6.81**
Study 16	**1.260**	**0.541**	**1.979**	**11.36**
Study 18	**0.510**	**-0.380**	**1.400**	**8.05**
Study 20	**1.010**	**0.079**	**1.941**	**7.46**
theta	**0.736**	**0.326**	**1.145**	
Group: 2				
Study 2	**0.134**	**-0.560**	**0.828**	**12.01**
Study 4	**0.947**	**0.108**	**1.786**	**8.88**
Study 12	**0.180**	**-0.426**	**0.786**	**14.68**
Study 14	**0.190**	**-0.416**	**0.796**	**14.68**
theta	**0.294**	**-0.040**	**0.628**	
Group: 3				
Study 8	**0.070**	**-0.910**	**1.050**	**6.83**
theta	**0.070**	**-0.910**	**1.050**	
Overall				
theta	**0.474**	**0.199**	**0.749**	

Heterogeneity summary

Group	df	Q	P > Q	tau2	% I2	H2
1	**4**	**4.13**	**0.389**	**0.028**	**12.58**	**1.14**
2	**3**	**2.78**	**0.427**	**0.000**	**0.00**	**1.00**
3	**0**	**0.00**	**.**	**0.000**	**.**	**.**
Overall	**9**	**10.59**	**0.305**	**0.039**	**19.96**	**1.25**

Test of group differences: Q_b = chi2(**2**) = **3.28** Prob > Q_b = **0.194**

```
5 . meta regress i.t, random(dl)
```

```
note: 4.t identifies no observations in the sample.
note: 5.t identifies no observations in the sample.

  Effect-size label: Effect size
        Effect size: smd
          Std. err.: se_smd

Random-effects meta-regression                    Number of obs  =        10
Method: DerSimonian-Laird                         Residual heterogeneity:
                                                              tau2 =       0
                                                            I2 (%) =    0.00
                                                                H2 =    1.00
                                                     R-squared (%) =  100.00
                                                  Wald chi2(2)   =      3.68
                                                  Prob > chi2    =    0.1588

------------------------------------------------------------------------------
    _meta_es | Coefficient  Std. err.      z    P>|z|     [95% conf. interval]
-------------+----------------------------------------------------------------
           t |
          2  |  -.4496384   .2587247    -1.74   0.082    -.9567295    .0574527
          3  |  -.6737772   .5365225    -1.26   0.209    -1.725342    .3777875
          4  |          0  (empty)
          5  |          0  (empty)
             |
       _cons |   .7437772   .1945671     3.82   0.000     .3624327    1.125122
------------------------------------------------------------------------------
Test of residual homogeneity: Q_res = chi2(7) =  6.91    Prob > Q_res = 0.4384

6 .
```
